# Evaluation of the Intraoperative Perfusion Index for Correlation with Acute Postoperative Pain in Patients Undergoing Laparoscopic Colorectal Cancer Surgery

**DOI:** 10.3390/jcm8091299

**Published:** 2019-08-24

**Authors:** Ji Hye Kwon, Hue Jung Park, Woo Seog Sim, Joo Hyun Park, Kang Ha Jung, Min Seok Oh, Heui Jin Seon, Jin Young Lee

**Affiliations:** 1 Department of Anesthesiology and Pain Medicine, Samsung Medical Center, Sungkyunkwan University, School of Medicine, Seoul 06351, Korea; 2 Department of Anesthesiology and Pain Medicine, Seoul St. Mary’s Hospital, College of Medicine, The Catholic University of Korea, Seoul 06591, Korea

**Keywords:** acute, change ratio, colorectal cancer, laparoscopic, perfusion index, postoperative pain

## Abstract

Despite technical advancements in the perioperative management of cancer surgery, postoperative pain remains a significant clinical issue. We examined the diagnostic value of the intraoperative perfusion index for predicting acute postoperative pain in patients undergoing laparoscopic colorectal cancer surgery. We retrospectively analyzed data for 105 patients who had undergone laparoscopic colorectal cancer surgery. Patients with pain scores <7 and ≥7 on a 10-point scale upon arrival in the postoperative anesthesia care unit (PACU) were categorized into the N and P groups, respectively. The perfusion index value was extracted prior to intubation, at the start and end of surgery, and after extubation. To minimize individual variance in the absolute value of the perfusion index, we calculated the perfusion index change ratio. A total of 98 patients were examined. Among them, 50 (51.0%) and 48 (49.0%) patients reported pain scores of <7 and ≥7 upon arrival at the PACU, respectively. Fentanyl consumption during the intraoperative and PACU periods was significantly higher in Group P than in Group N (*p* < 0.001). The perfusion index change ratios did not significantly differ between the groups. The intraoperative perfusion index change ratios do not correlate with acute postoperative pain following laparoscopic colorectal cancer surgery.

## 1. Introduction

Despite technical advancements in the perioperative management of cancer surgery, postoperative pain remains a significant clinical issue [1]. Previous studies have reported that the incidence of moderate to severe postoperative pain ranges from 12% to 80% [2,3]. Pain caused by surgical procedures impacts postoperative rehabilitation and quality of life [3,4,5]. Because surgical incisions activate peripheral and central sensitizations, the patient’s pain status should be identified early to prevent persistent postoperative pain [6]. However, there are no objective methods for assessing intraoperative nociception [7]. Korhonen and colleagues [8] reported that skin vasomotor responses and photoplethysmography amplitudes are associated with nociception during general anesthesia. However, this association may be affected by several confounding factors such as physiological variables (e.g., hypothermia, hypovolemia, and circulatory conditions) or pharmacological treatments (e.g., anesthetics, analgesics, and vasoactive drugs) [8]. The perfusion index (PI), which refers to the ratio of the alternating current component to the direct current component, is a quantitative representation of the photoplethysmography waveform that reflects real-time changes in peripheral blood flow at the site being monitored [9]. Tapar and colleagues [10] evaluated the utility of PI values for assessing postoperative pain and responses to analgesics in the recovery room. However, no previous studies have tested for a correlation between PI values and intraoperative nociception. In the present study, we examined whether the intraoperative PI values correlate with acute postoperative pain levels in patients undergoing laparoscopic colorectal cancer surgery. We focused on colorectal cancer surgery for this study because it may induce complex postoperative pain, including somatic and visceral pain from surgical incisions affecting intra-abdominal structures and neuropathic pain due to stimulation of the pelvic plexus [11].

## 2. Materials and Methods

### 2.1. Patients

We retrospectively reviewed the electronic medical records of patients who underwent elective laparoscopic colorectal surgery from January 2019 to April 2019 at a single center. The patients ranged in age from 28 to 82 years. All patients had tumors within the colon or rectum and had undergone laparoscopic colectomy or anterior resection. The exclusion criteria were as follows: preoperative pain therapy, emergent surgery, recurrent colorectal lesions, metastatic colorectal lesions, conversion to open surgery, a lack of follow-up data, need for intensive care unit management after surgery, and inability to express pain severity. This study was approved by our departmental ethics committee (ref: SMC 2019-06-047) and registered with Clinical Research Information Service of the Korea National Institute of Health, ref: KCT0004096, (http://cris.nih.go.kr/cris/index.jsp). 

### 2.2. Intervention

Patients were not preoperatively medicated. Intraoperative anesthetic management was standardized. Standard monitoring (IntelliVue MP70, Philips Healthcare, Best, Netherlands) was performed and included oxygen saturation, electrocardiography, end-tidal carbon dioxide, pulse oximetry, bispectral index (BIS), and non-invasive blood pressure measurements. Anesthesia was induced intravenously with 40 mg of 2% lidocaine, 2 mg/kg of 2% propofol, 0.5–1 µg/kg of fentanyl, and 0.6–0.8 mg/kg of rocuronium. Endotracheal intubation was performed using a Macintosh laryngoscope after approximately 3–5 min of mask ventilation and the loss of all four twitches following train-of-four stimulation of the ulnar nerve. After tracheal intubation, anesthesia was maintained with 1.5–3.0 vol. % sevoflurane and a bolus injection of 0.5–1 µg/kg of fentanyl to maintain hemodynamic parameters within 20% of baseline values and a BIS of 40–60. The lungs were ventilated with 50% oxygen with air. This was adjusted to maintain an end-tidal carbon dioxide level of 30–40 mmHg. Body temperature was controlled at a target value of 36.5 °C. During anesthesia, the PI was continuously monitored via pulse oximetry (Root^®^, Mashimo Corporation, Irvine, CA, USA) of the index finger. It was placed on the hand contralateral to the side of the blood pressure cuff. All surgeries were performed by one of six specialized colorectal surgeons who followed similar techniques for colorectal cancer. Approximately 20 min before the end of surgery, patients received an intravenous patient-controlled analgesia pump (Automed3200^®^, Ace Medical, Korea), which delivered 25 µg/kg of fentanyl in normal saline (100 mL) at a basal infusion rate of 0.5 mL/h and a bolus of 1 mL. The lock out interval was 15 min. At the end of surgery, 0.03 mg/kg of intravenous pyridostigmine and 0.002 mg/kg of intravenous glycopyrrolate were administered to the patients. After extubation, patients were moved to the postoperative anesthesia care unit (PACU). They received a further bolus of intravenous fentanyl at 0.5 µg/kg when visual analogue scale (VAS, 0 = no pain; 10 = intolerable pain) scores were greater than 3 [12]. Pain scores were recorded before anesthesia, upon arrival at the PACU, and at discharge. Patients with pain scores <7 and ≥7 upon arrival at the PACU were categorized into the N and P groups, respectively.

### 2.3. Statistical Analysis

All data were analyzed using SAS 9.4 (SAS Institute, Cary, NC, USA). Data are expressed as means ± standard deviations (SD) or numbers (with percentages), as appropriate. Demographic and clinical data for the two groups were compared using a Chi-square test, a t-test, or Fisher’s exact test. We extracted PI values prior to anesthesia (T1), at the surgical incision (T2), at the end of surgery (T3), and after extubation (T4). To minimize individual variance in the absolute values of the PI and BIS, we calculated PI change ratios (PI at each time point − PI at T1/PI at T1) and BIS change ratios (BIS at each time point − BIS at T1/BIS at T1) at T2, T3, and T4. These ratios were compared using t-tests. In each group, differences in PI and BIS values over time were compared using a generalized estimating equations (GEE) analysis. The level of statistical significance was set at *p* < 0.05.

## 3. Results

**Figure 1:**
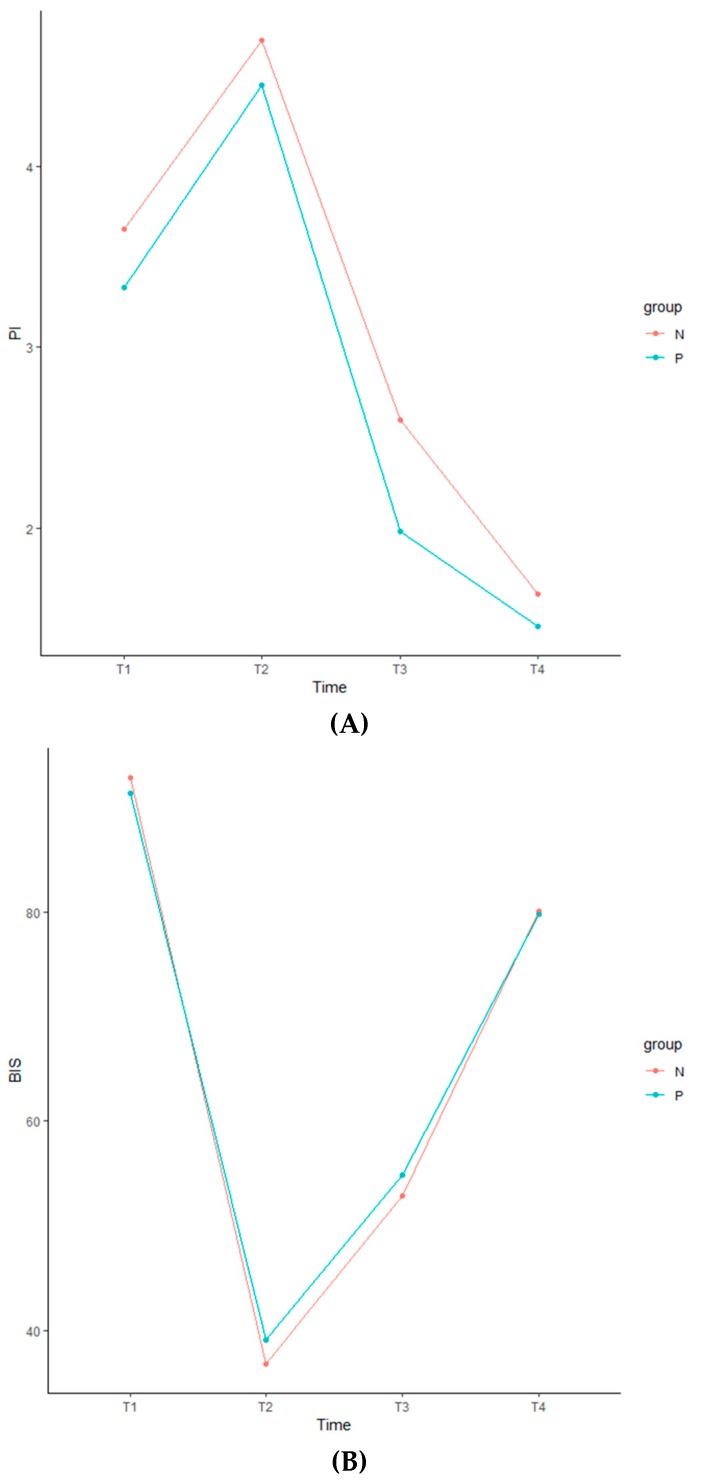
Perfusion index (**A**) and bispectral index (**B**) during anesthesia. We extracted values prior to anesthesia (T1), at surgical incision (T2), at the end of surgery (T3), and after extubation (T4). Group N: patients with pain scores <7 in the PACU, Group P: patients with pain scores ≥7 in the PACU. PI: perfusion index, BIS: bispectral index, PACU: postoperative anesthesia care unit.

Of the 105 patients assessed for eligibility, seven were excluded due to insufficient medical records. Thus, data were analyzed for a total of 98 patients. Demographic and clinical data are summarized in Table 1. Age, sex, body mass index, American Society of Anesthesiologists status, diagnosis, presence of diabetes mellitus, preoperative chemoradiotherapy, surgery type, pathological stage, mean tumor size, operation and anesthesia times, and preoperative pain scores did not significantly differ between the two groups (Table 1). Fentanyl consumption during the intraoperative and PACU periods was significantly higher in Group P than in Group N. The PI and BIS change ratios are presented as the means ± SDs (Table 2). There were no significant differences in PI or BIS change ratios over time (from T2 to T4) between the two groups (Table 2). The GEE analysis revealed no significant between-group differences in PI values (*p* = 0.803) or BIS values (*p* = 0.222) at any time point (Table 3). In both groups, PI and BIS values significantly differed at each time point (from T1 to T4) (Figure 1), (Table 3). The data are expressed as means ± SDs (Table 4).

## 4. Discussion

In the present study, we aimed to determine whether intraoperative PI values correlate with acute postoperative pain in patients undergoing laparoscopic colorectal cancer surgery. However, there were no significant differences in PI change ratios over time between patients with pain scores <7 and those with pain scores ≥7.

During anesthesia, balanced analgesia is critical for reducing postoperative pain and analgesia related side effects [13]. Under- or over-treatment of postoperative pain leads to increased morbidity and mortality, increased lengths of hospital stays, and the development of chronic pain [14]. However, clinical monitoring of intraoperative analgesia remains challenging. Despite the introduction of numerous devices, none have sufficient sensitivity and specificity. Monitoring of intraoperative nociception is currently based on hemodynamic or electroencephalic analyses. Previous studies have utilized the analgesia nociception index, skin conductance, pupillometry, nociceptive flexion reflex thresholds, the surgical pleth index, and the nociception level index [15,16,17]. These measurements can reflect sympathetic and parasympathetic tone, as surgical or other noxious stimuli lead to vasomotor-related changes [8,18]. However, sympathetic stress responses do not exhibit a linear association with noxious stimuli and may be unpredictable due to the use of intraoperative drugs, differences in intravascular fluid status, differences in the type of general anesthesia, differences in autonomic tone alterations due to age, and the use of opioids and/or neuromuscular blockers that induce changes in pupillary diameter [16]. In major abdominal surgery, use of the nociception level index reduced intraoperative remifentanil use by 30% but had no effect on postoperative pain scores or postoperative opioid requirements [15]. The analgesia nociception index can be used to detect surgical stimuli in children, although no studies have evaluated the correlation between this index and postoperative pain or opioid consumption [17]. The PI is a ratio between pulsatile and non-pulsatile signals that reflects peripheral perfusion [19,20]. When the sympathetic nervous system is activated, the PI may decrease due to increased vasomotor tone and contraction of peripheral blood vessels [21]. The PI has recently received greater attention in the fields of regional anesthesia and chronic pain diagnosis and management [10,21,22,23]. Mowafi and colleagues [18] demonstrated that the PI is a reliable measure for the intravascular injection of epinephrine during epidural anesthesia. Some reports have indicated that the PI can be used for pain assessment in patients with critical illnesses, at the onset of stellate ganglion block, in lumbar and thoracic sympathectomy, or for predicting the success of brachial plexus, sciatic, or lumbar transforaminal blocks [20,23,24,25,26,27].

In the present study, intraoperative PI change ratios did not correlate acute postoperative pain. PI values significantly increased following the induction of anesthesia due to decreased sympathetic tone. These values decreased following surgical incisions due to increased sympathetic tone and decreased further after extubation due to acute postoperative pain, anxiety, or agitation. BIS values exhibited an inverse pattern when compared with PI values. Because there were no significant differences in PI or BIS change ratios and no relationship between mean PI and BIS values over time, the depth of consciousness might not be related to the PI during anesthesia. We suspect that general anesthesia agents may overcompensate for intraoperative sympathetic changes due to surgical stimuli, which may influence changes in PI values. Alternatively, differences in the pathophysiology of acute and chronic pain may explain the poor predictive capacity of intraoperative PI values. Chronic pain may be related to autonomic disturbances, as previous research has indicated that PI values are associated with improvements in pain after lumbar transforaminal block in patients with chronic lower radiculopathy [23]. In a rat model of chronic back pain, subjects exhibited decreased renal blood flow due to altered sympathetic regulation as well as structural changes in the cingulate cortex, both of which may be related to sympathetic dysregulation [28]. However, acute somatic and/or visceral pain cannot be measured simply based on sympathetic changes during anesthesia. Furthermore, other factors affecting postoperative pain cannot be explained based on sympathetic changes alone. Indeed, intraoperative nerve injury, tissue ischemia, interpersonal pain facilitation or amplification, and inflammatory states should be considered as causes of postoperative pain [29,30,31].

This study had several limitations. First, we did not measure PI values in the PACU due to poor patient cooperation. PI measurements are quite sensitive to patient, probe, and tissue movements, which may cause rapid fluctuations in PI values. Second, we did not rule out preoperative psychosocial factors including anxiety, depression, sleep disturbances, and stress [29]. Third, we selected a VAS of 7 as the cutoff value for categorizing postoperative pain levels, and scores ≥7 probably correspond to severe postoperative pain rather than mild or moderate postoperative pain.

## 5. Conclusions

The results of the present study suggest that PI change ratios do not correlate with acute postoperative pain in patients undergoing laparoscopic colorectal surgery. Future prospective randomized studies are required to determine whether more targeted parameters can provide superior diagnostic value for nociception under anesthesia.

## Figures and Tables

**Table 1:** Demographic and clinical characteristics of the included patients.

	All Patients (*n* = 98)	Group N (*n* = 50)	Group P (*n* = 48)	*p*-Value
Age (years)	62.9 ± 12.8	65.3 ± 13.2	60.5 ± 11.9	0.064
Sex (Male/Female)	50/48	25/25	25/23	0.997
Body mass index (kg/m^2^)	24.0 ± 3.2	23.8 ± 3.2	24.3 ± 3.1	0.436
ASA status: I/II/III	39/52/7	19/27/4	20/25/3	0.952
Diagnosis				0.098
Colon cancer	69 (70.4%)	32 (64.0%)	37 (77.1%)	
Rectal cancer	29 (29.6%)	18 (36.0%)	11 (22.9%)	
Presence of DM	16 (16.3%)	8 (16.0%)	8 (16.7%)	1.000
Preoperative CRT	10 (10.2%)	3 (6.0%)	7 (14.6%)	0.195
Surgery type				
Colectomy	54 (55.1%)	27 (54.0%)	27 (56.3%)	0.599
Low anterior resection	44 (44.9%)	23 (46.0%)	21 (43.7%)	
Pathological stage				
I	26 (26.5%)	14 (28.0%)	12 (25.0%)	0.673
II	19 (19.4%)	7 (14.0%)	12 (25.0%)	
III	11 (11.2%)	6 (12.0%)	5 (10.4%)	
IV	42 (42.9%)	23 (46.0%)	19 (39.6%)	
Mean tumor size > 4 cm	57 (58.2%)	26 (52.0%)	31 (64.6%)	0.290
Operation time (min)	144.4 ± 58.4	137.7 ± 43.8	151.3 ± 70.3	0.259
Anesthesia time (min)	190.7 ± 60.2	185.3 ± 45.1	196.3 ± 72.8	0.374
Intraoperative fentanyl (µg)	59.3 ± 23.9	54.4 ± 22.8	64.3 ± 24.7 *	0.039
Preoperative pain (VAS)	0.1 ± 0.6	0.0 ± 0.4	0.2 ± 0.7	0.379
Postoperative pain at PACU				
Admission	6.4 ± 1.8	4.9 ± 1.2	7.8 ± 1.0 *	<0.001
Discharge	2.9 ± 0.8	2.7 ± 0.6	3.0 ± 0.9 *	0.026
Rescue fentanyl at PACU (µg)	53.8 ± 31.2	42.6 ± 26.7	65.5 ± 31.4 *	<0.001

All data are presented as means ± SDs or numbers (percentages) of patients. ASA: American Society of Anesthesiologists, DM: diabetic mellitus, CRT: chemoradiotherapy, VAS: visual analogue scale, PACU: postoperative anesthesia care unit; Group N: patients with pain scores <7 in the PACU; Group P: patients with pain scores ≥7 in the PACU, * *p* < 0.05 was considered statistically significant.

**Table 2:** Perfusion index and bispectral index change ratios over time.

	Group N (*n* = 50)	Group P (*n* = 48)	*p*-Value
PI change ratio			
T2	0.98 ± 1.7	1.02 ± 1.8	0.909
T3	0.00 ± 1.0	−0.12 ± 1.3	0.635
T4	−0.36 ± 0.62	−0.40 ± 0.5	0.729
BIS change ratio			
T2	−0.60 ± 0.1	−0.55 ± 0.2	0.123
T3	−0.43 ± 0.1	−0.36 ± 0.3	0.181
T4	−0.13 ± 0.1	−0.13 ± 0.1	0.786

PI: perfusion index, BIS: bispectral index, T2: start of surgery, T3: end of surgery, T4: after extubation, PI change ratio (PI at each time point − PI at T1/PI at T1), BIS change ratio (BIS at each time point − BIS at T1/BIS at T1), Group N: patients with pain scores <7 in the PACU, Group P: patients with pain scores ≥7 in the PACU, PACU: postoperative anesthesia care unit, *p* < 0.05 was considered statistically significant.

**Table 3:** Analysis of generalized estimating equations.

Parameter	Estimate	SE	95% Confidence Limits		*p*-Value
PI values					
Intercept	5.1413	0.4850	4.1906	6.0919	<0.0001
Time	−0.8938	0.1457	−1.1793	−0.6082	<0.0001
Group	0.5059	0.8001	−1.0623	2.0741	0.5272
Time, group	−0.0544	0.2184	−0.4824	0.3737	0.8034
BIS values					
Intercept	70.7969	1.4266	68.0009	73.5929	<0.0001
Time	−1.3688	0.6316	−2.6066	−0.1309	0.0302
Group	0.4888	2.0224	−3.4750	4.4527	0.8090
Time, group	−1.1491	0.9412	−2.9938	0.6956	0.2221

PI: perfusion index, BIS: bispectral index, SE: standard error, *p* < 0.05 was considered statistically significant.

**Table 4:** Perfusion index and bispectral index values over time.

	Group N (*n* = 50)	Group P (*n* = 48)
PI values		
T1	3.65 ± 2.7	3.33 ± 2.3
T2	4.69 ± 2.3	4.45 ± 2.1
T3	2.60 ± 2.1	1.98 ± 1.8
T4	1.64 ± 1.3	1.46 ± 1.1
BIS values		
T1	92.84 ± 5.1	91.29 ± 11.9
T2	36.78 ± 8.5	39.04 ± 10.4
T3	52.84 ± 10.6	54.79 ± 10.9
T4	80.04 ± 9.5	79.73 ± 13.4

All data are presented as means ± SDs. PI: perfusion index, BIS: bispectral index, T1: before intubation, T2: start of surgery, T3: end of surgery, T4: after extubation, Group N: patients with pain scores <7 in the PACU, Group P: patients with pain scores ≥7 in the PACU, PACU: postoperative anesthesia care unit.
